# Benchmarking the Humidity-Dependent Mechanical Response of (Nano)fibrillated Cellulose and Dissolved Polysaccharides as Sustainable Sand Amendments

**DOI:** 10.1021/acs.biomac.3c01294

**Published:** 2024-03-08

**Authors:** M-Haidar A. Dali, Roozbeh Abidnejad, Mohamed Hamid Salim, Mamata Bhattarai, Monireh Imani, Orlando J. Rojas, Luiz G. Greca, Blaise L. Tardy

**Affiliations:** † Department of Chemical Engineering, Khalifa University, Abu Dhabi, United Arab Emirates; ‡ Research and Innovation Center on CO_2_ and Hydrogen, Khalifa University, Abu Dhabi, United Arab Emirates; § Department of Bioproducts and Biosystems, School of Chemical Engineering, Aalto University, P.O. Box 16300, FI-00076 Aalto, Finland; ∥ Center for Membrane and Advanced Water Technology, Khalifa University, Abu Dhabi, United Arab Emirates; ⊥ Bioproducts Institute, Department of Chemical and Biological Engineering, Department of Chemistry and Department of Wood Science, University of British Columbia, 2360 East Mall, Vancouver, BC V6T 1Z4, Canada; # Laboratory for Cellulose & Wood Materials, Empa—Swiss Federal Laboratories for Materials Science and Technology, Überlandstrasse 129, 8600 Dübendorf, Switzerland

## Abstract

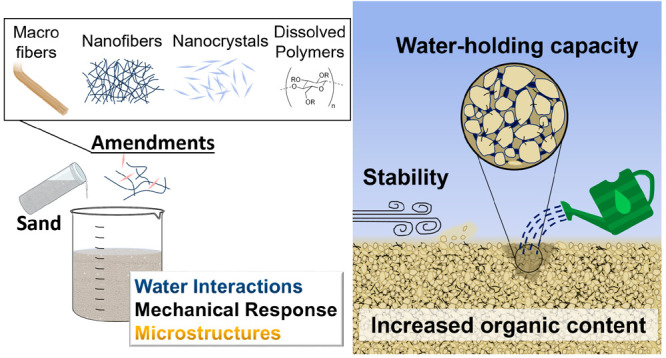

Soil quality is one of the main limiting factor in the development of the food sector in arid areas, mainly due to its poor mechanics and lack of water retention. Soil’s organic carbon is nearly absent in arid soils, though it is important for water and nutrient transport, to soil mechanics, to prevent erosion, and as a long-term carbon sink. In this study, we evaluate the potential benefits that are brought to inert sand by the incorporation of a range of, mainly, cellulosic networks in their polymeric or structured (fiber) forms, analogously to those found in healthy soils. We explore the impact of a wide range of nonfood polysaccharide-based amendments, including pulp fibers, nanocellulose, cellulose derivatives, and other readily available polysaccharide structures derived from arthropods (chitosan) or fruit peels (pectin) residues. A practical methodology is presented to form sand–polymer composites, which are evaluated for their soil mechanics as a function of humidity and the dynamics of their response to water. The mechanics are correlated to the network of polymers formed within the pores of the sandy soil, as observed by electron microscopy. The response to water is correlated to both the features of the network and the individual polysaccharides’ physicochemical features. We expect this work to provide a rapid and reproducible methodology to benchmark sustainable organic amendments for arid soils.

## Introduction

Fertile soils are a foundational element for human survivability on Earth.^1^ Soils are not only a resource for food, biomass, and fiber production, but they also provide crucial services for the ecosystem, like water purification, and work as a major carbon sink.^2−5^ Using and improving soils’ carbon reservoirs is associated with other benefits, for instance, the growth of the food sector and the development of greeneries beneficial to overall wellbeing.^6^ These developments are particularly challenging in arid areas, which cover over 50 million km^2^. Arid soils contain a very low organic content, presenting a high risk of erosion, low water retention capacity, and thus an overall low potential for agriculture.^7^ Without interventions, in the coming decades, arid areas are expected to increase substantially,^8^ and significant efforts are being made to reduce the further spread of arid areas, for instance, by means of consolidating the granular soil and increasing its organic content.^9−12^ There is a pressing need to improve the overall cohesion of desertic soils with the end goal of improving their potential for plant growth and cropping. Such strategies must consider both localized sourcing that lower transportation and minimally exhaustive processes that prevent the generation and release of environmental hazards.^13,14^ These are crucial aspects to optimize the overall sustainability of the approach and its durability over periods spanning decades or longer.

Cohesive interactions in conventional soil are typically associated with its clay content and primarily with the presence of a high fraction of organic matter.^15,16^ Organic amendments have been used to maintain the quality of soils for as long as agricultural activities are present in historical records, e.g., in the form of manure, compost, or charred biomass. While the presence of clay depends on its natural occurrence, the presence of organic matter in soils can be correlated directly with agriculture, which generates its own organic amendments.^17^

Polysaccharides are one of the most important classes of organic matter in soils as they can enhance multiscaled water interactions,^18^ enhance the cohesion of granulated matter, serve as nutrients for the microbiome,^3^ and eventually act as a long-term sink of carbon.^19^ In recent years, the use of natural polymers as soil amendments has been extensively studied, showcasing their potential benefits.^12,20−22^ Gums have been the most explored carbohydrate polymers to improve the cohesive properties of soils that are otherwise unfit for agriculture or highly prone to erosion.^20^ While they present excellent properties, there are alternative sources that do not contribute to the food supply chain,^23−25^ such as basic and advanced cellulosics, which may bring the largest benefits as amendments due to their wider spread availability and inherent properties.

Cellulosics, constituting 40–50% of global biomass, represent the most abundant class of polysaccharides.^26^ Each year, approximately 13 billion metric tons of biomass is generated, posing a growing concern for effective management.^27^ They are nonfood and nonfeed and therefore may represent the ideal building block for soil amendment.^24^ Furthermore, cellulose is one of the most resilient natural polymers, as it is highly stable under chemical, heat, and saline stresses. In the past decade, cellulosics have been enriched substantially with the many forms of micro- and nanoscaled celluloses presently available.^28,29^ However, their potential to improve the mechanical properties and water interaction of sandy and granular soils remains to be systematically explored.

**Figure 1:**
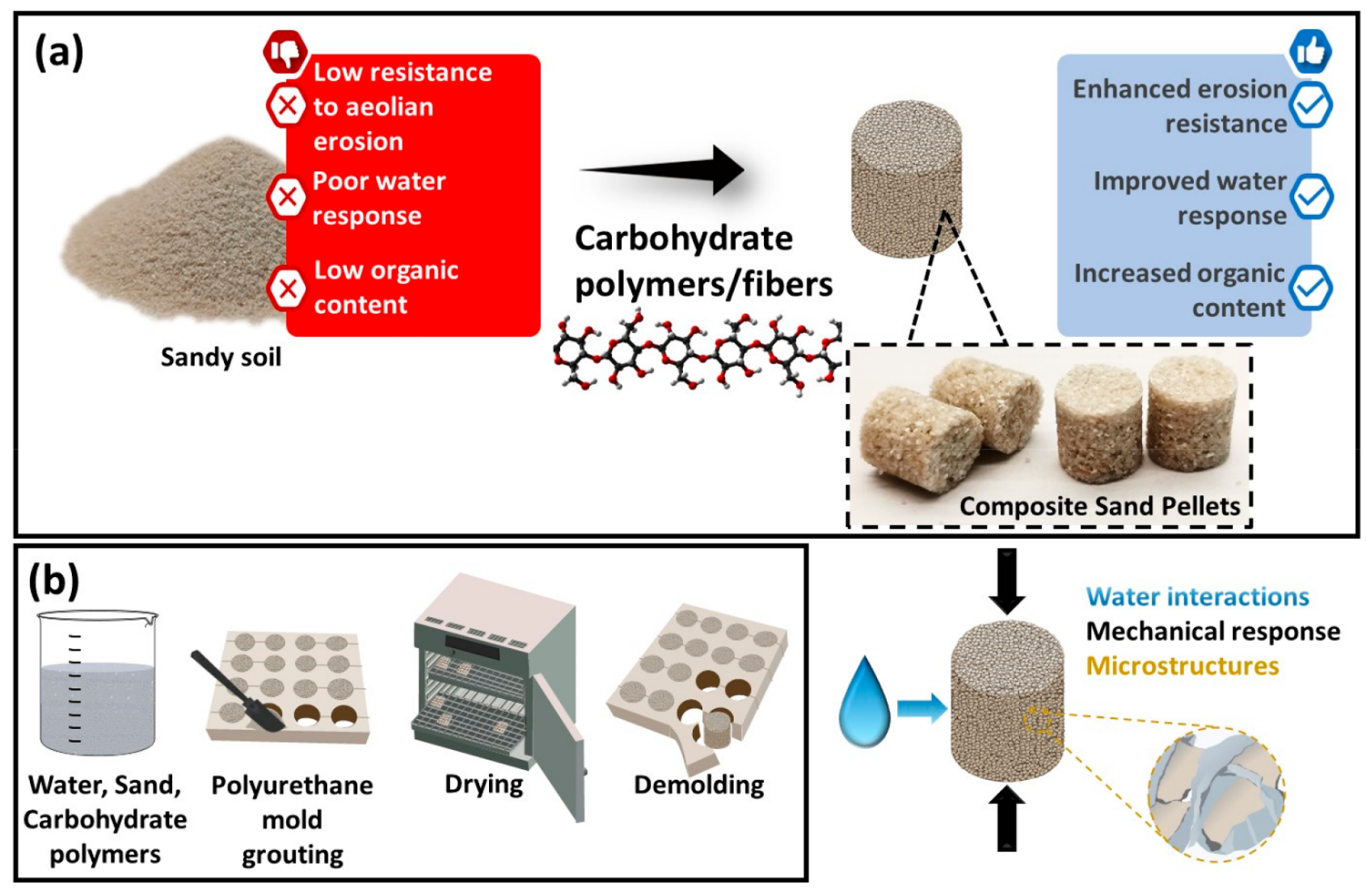
(a) Impact of polysaccharides and their fibers as enhancers of sandy soils. (b) Preparation of composite sand pellets for testing of mechanical properties and water-dependent response, as described in the right-hand side panel.

Herein, the potential of a wide range of polysaccharides is systematically assessed for their potential to address the key issues of conventional sand (Figure 1a). A particular focus is put on cellulose derivatives, pulp fibers, and a range of nanocelluloses. A simple and rapid approach is put forward (Figure 1b) to benchmark the mechanical, humidity-dependent performance of cellulose derivatives as well as nanocelluloses and also include three highly abundant polysaccharides—namely, alginate, pectin, and chitosan. Our findings reveal distinct responses to compression stresses among these polysaccharides, which can be associated with their physicochemical characteristics. The latter results in various polymeric networks and grain clusters’ sizes, which are linked to 3D networks formed within packed particles.^30^ The internal microstructures formed between grains for three selected polysaccharides are also evaluated using scanning electron microscopy, and their impact on water interactions is evaluated by dynamic water vapor sorption. The importance of the surface charge of the amendments is also evaluated for polysaccharides with pH-dependent charges at key pH values (well above or below the acid dissociation constants). Finally, the impact of the concentrations of selected polymeric and nanofibrous systems was thoroughly evaluated. Overall, we expect the study to provide deeper insights into the potential of cellulosics to improve the mechanics and humidity response of granular inorganic materials, such as sandy soils. This research explores aspects of nanocellulose applications in this new context—including impact of humidity, pH effects, and fracture regimes—in a field that has seen substantial attention and growth in recent times.^12,20−22^

## Materials and Methods

### Materials

Mechanically fibrillated cellulose nanofibers (CNFs) were prepared by mechanical disintegration from never-dried, fully bleached, and fines-free sulfite birch pulp (Kappa number of 1, DP of 4700) suspended in distilled water at 1.8% (w/v). The suspension was disintegrated using a high-pressure fluidizer (Microfluidics M110P, 6, 9, or 12 passes). Cellulose nanocrystals were obtained through the Process Development Center, University of Maine, and produced by Forest Products Laboratory (FPL, Madison, WI) affiliated with the United States Department of Agriculture.^31^ Tempo-oxidized fibrils were obtained from the same precursor as the mechanically fibrillated CNFs using a protocol previously described.^29^ Besides fibers, all polymers were obtained commercially from Sigma-Aldrich, and their additional characteristics were reported across studies reported by the supplier. The sodium salt of carboxymethyl cellulose (CMC, molecular weight 0.7 MDa and 0.09 MDa), methylcellulose (viscosity: 15 cP), HPC (hydroxypropyl cellulose, MW 100 kDa, DS 2.2^32^), sodium alginate (molecular weight, 12–40 kDa), chitosan (medium molecular weight, 190–310 kDa), pectin (from citrus, whole fractions with galacturonic acid ≥74.0% (dried basis), CAS Number: 9000-69-5), sodium hydroxide pellets, and hydrogen chloride (37%) were purchased from Sigma-Aldrich. Kraft pulp (KP, once dried) and bleached kraft pulp (BKP, never dried) from Finnish birch were obtained from a Finnish mill (UPM). For simplicity, these diverse polysaccharide-based additives are generically termed “amendments”. Commercial quartz granules (mesh size of 50–70) were obtained from Sigma-Aldrich.

### Preparation of Sand Pellets

Aqueous suspensions, or solutions, of 2.5 wt % of the given amendments were initially prepared with distilled water. In the case of chitosan, due to low solubility, the polymer powder was briefly gelled with a small amount of 1 M HCl solution, and then the concentration was adjusted to ∼5 wt % followed by adjusting the pH and concentration to 5 and 2.5 wt %, respectively.

A fixed weight of 14 g of sand was placed into a container, and the various amendments (equivalent to 2.5 wt % or 6 g of a solution or suspension) were introduced in two distinct manners: First, for the data showcased in Figure 6, when the dispersion exhibited high viscosity to prevent air bubble formation, the amendments were infiltrated into the packed sand using centrifugation. Second, the data presented in Figure 7, which involved amendments such as fibers, were mixed into a slurry with rigorous mixing. The slurry was then gently mixed with a spatula and carefully grouted into cylindrical 3D-printed thermoplastic polyurethane molds with a diameter of 6 mm and a height of 6 mm. The final pellets weighed approximately 210 mg and contained 1.06% of the polymer by weight unless specified otherwise. 3D-printed polyurethane molds were used for their temperature resistance and flexibility, which enabled facile isolation of formed composites with minimal demolding stresses applied to the samples. In the case of pH-modified samples (CMC_0.7_ and chitosan), after forming the pellets in the molds, they were subsequently immersed for more than 3 h in 0.1 M NaOH or HCl followed by three immersions in deionized water for over 3 h to remove excess base or acid. No loss of sand was observed, which suggested that the cohesion was not affected by the immersion steps. After being dried for 4 h at 105 °C, the molds were opened, and the consolidated composite cylinders were collected. Typically, it was possible to collect as many as 16 samples per mold. However, for less robust samples, such as suboptimal polymers such as pectin, as few as 3 out of 16 samples could be obtained.

To identify the most effective drying method for subsequent mechanical analysis, the effect of temperature and drying kinetics was evaluated on CMC_0.7_ (Figure S1). It was shown that polymer-reinforced sand pellets dried at 40 °C had more strength than those dried at 105 °C. However, the mechanical characteristics of the pellets were not reduced when dried at 40 °C and then exposed to the same drying cycle at 105 °C. This suggests that the observed enhancement in mechanical properties for the samples dried at 40 °C was due to the drying rate as opposed to thermal stability; i.e., the pellet structure was preserved after the polymer–grain interaction was enabled by the slower drying rate.

It is important to note that for both macro- and nanofibrous samples, the concentrations were adjusted to six times the original value for KP (designated as KP 6X) and two times the original value for both BKP (BKP 2X) and CNFs (CNF 2X). This adjustment was made because initially we did not achieve a continuous network of fibers at the same concentrations as those used for evaluating the dissolved polymers, which was necessary to consolidate the sand samples into cylinders. Consequently, these adjusted data sets were excluded from the quantitative analysis presented in Figure 7. It is worth mentioning that the difference between BKP and KP is due to the fact that BKP underwent no drying process, while KP was subjected to a single drying cycle.

### Compression Tests

Axial compression tests were conducted using a TA.XTplusC texture analysis instrument (Stable Micro Systems, Godalming, UK) for the data shown in Figure 6, while for the data displayed in Figures 7, S1, and S5, an Instron 5948 micromechanical tester was employed. The cylindrical samples were positioned vertically between two parallel steel plates. The strain rate was set to 0.2 mm s^–1^, and the measurement was interrupted at 50% strain. The stresses were calculated based on the (circular) cross-sectional area of the cylinder. The maximum load was used to evaluate the strength and the toughness was calculated over a strain of 25%. The samples were left to equilibrate for at least 1 day at 25% relative humidity (RH) or at saturated humidity (>95% RH), and their compression response was measured immediately afterward at 25% RH. Typically, at least 5 samples were tested for each condition.

### Scanning Electron Microscopy Imaging

Scanning electron microscopy (SEM) was used to image representative samples by using a Zeiss Sigma VP device with a Schottky field emission source. The samples were coated with a 4 nm thick layer of a platinum/palladium alloy.

### Dynamic Vapor Adsorption Measurements

The sorption/desorption isotherms were evaluated by dynamic vapor sorption (DVS Intrinsic, Surface Measurement Systems, UK). Nitrogen flow and temperature were kept constant at 200 sccm and ca. 25 °C, respectively. Prior to measurement, samples were dried at 105 °C. Then, approximately 20 mg of the sample was placed inside the apparatus. Both uptake and release isotherms were determined by conditioning the samples through set relative humidity stages. Each sample was evaluated three times.

## Results and Discussion

Polysaccharides exhibit diverse interactions with sandy soils, leading to altered fracture regimes, unique polymeric network formation, and particle cluster size variations. In this study, chitosan, carboxymethylcellulose with a *M*_W_ of 0.7 MDa and 0.09 MDa (CMC_0.7_ and CMC_0.09_, respectively), hydroxypropylcellulose (HPC), pectin, sodium alginate, mechanically fibrillated cellulose nanofibers (CNFs) with varying fibrillation severity, elementary fibrils (tempo-oxidized CNF or TO-CNF), cellulose nanocrystals (CNCs), bleached kraft pulp (BKP), kraft pulp (KP), and methylcellulose were evaluated for their ability to introduce cohesion to granular materials, i.e., quartz-rich sand (herein termed sand). The impact of pH was also evaluated for chitosan and CMC due to their weak polyelectrolyte nature, i.e., pH-dependent charges and associated water swelling at low and high pH, respectively. In the case of certain polymers, sodium alginate, for instance, the polymer migrated to the upper drying front and formed consolidated discs with unconsolidated portions underneath, and thus, the samples were not considered further (Figure S2).

**Figure 2:**
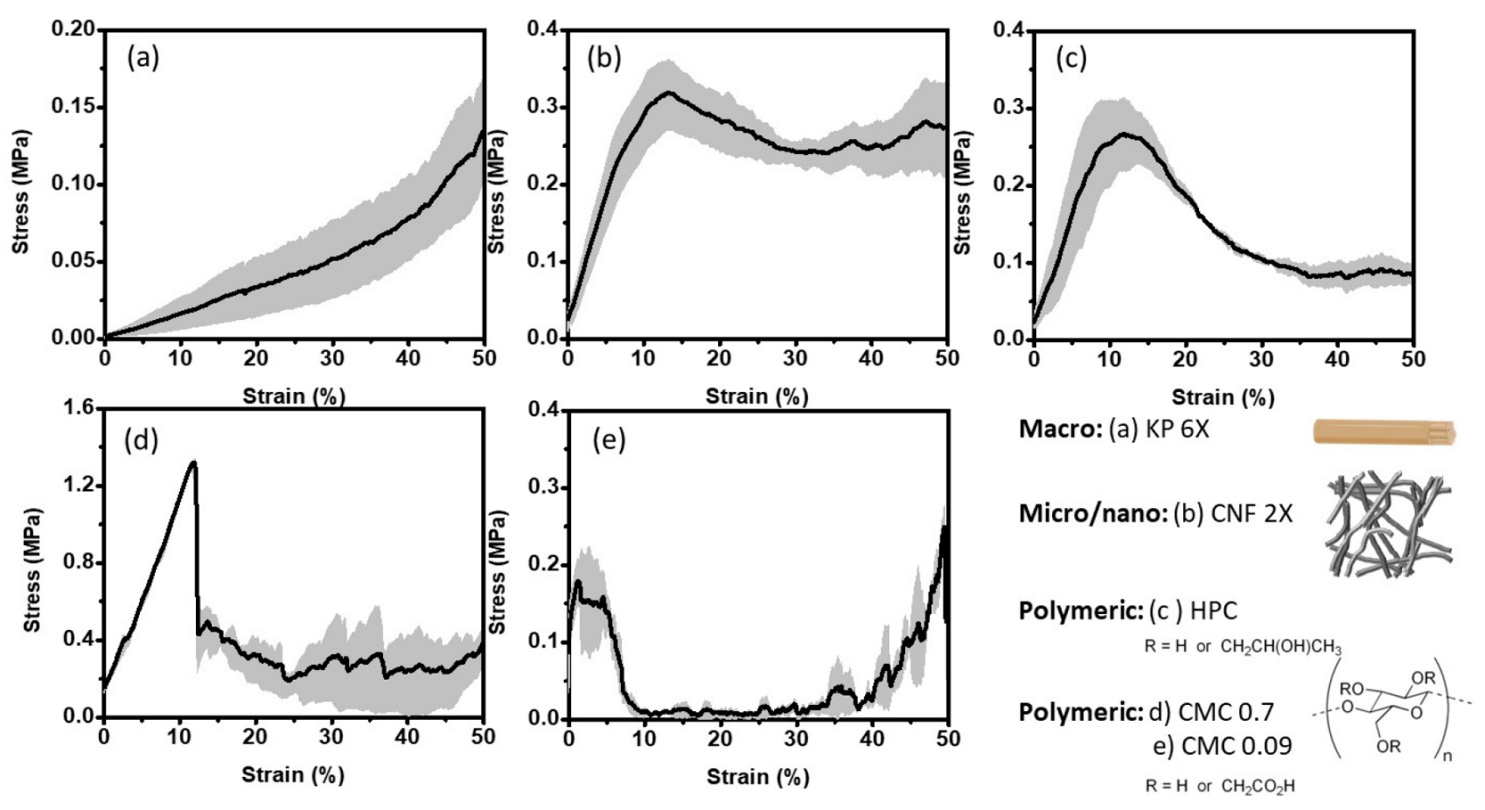
(a–e) Representative stress–strain curves at 25% RH for (a) KP, (b) CNF 2X, (c) HPC, (d) CMC_0.7_, and (e) CMC_0.09_ of unconfined compression tests for pellets formed with various carbohydrate polymer amendments (indicated next to (e)).

For the polymers that enabled the formation of consolidated pellets, a series of compression tests were performed. Representative curves are presented in Figure 2, enabling qualitative analysis of the fracturing/failure behaviors. As expected, the behavior was not elastic in most cases due to the brittle nature of dried polysaccharides. Three distinct morphologies of the tested material were observed at the end of the test, i.e., at 50% strain, and three different classes of responses to compression were identified (Figure S3), namely, (i) plastic deformation, i.e., top-side widening or buckling, both without fragmentation (Figure 2a), (ii) fracture at low strain into large fragments that sustain a continuous stress release (Figure 2b,c), and (iii) fracture at low strain followed by complete fragmentation into smaller aggregates as well as individual grains (Figure 2d,e). The fracture behaviors are associated with the structures formed during consolidation that affect the relative contribution of the polymer–polymer and polymer–sand interactions.^30,33^ The exact contributions of each component to the fractures are generally a complex combination of the two aforementioned interactions, as well as the overall network micro- and macrostructures. However, dominant mechanisms can be hypothesized as associated with stress–strain curve types and previous evidence in the literature, for example, as has been thoroughly studied in the case of nanofibers.^30^ For example, nanofibrillar carbohydrates present substantial sand–fiber slippage when compared with fiber–fiber adhesion if their network does not present a high degree of long-range order.^30,33^

**Figure 3:**
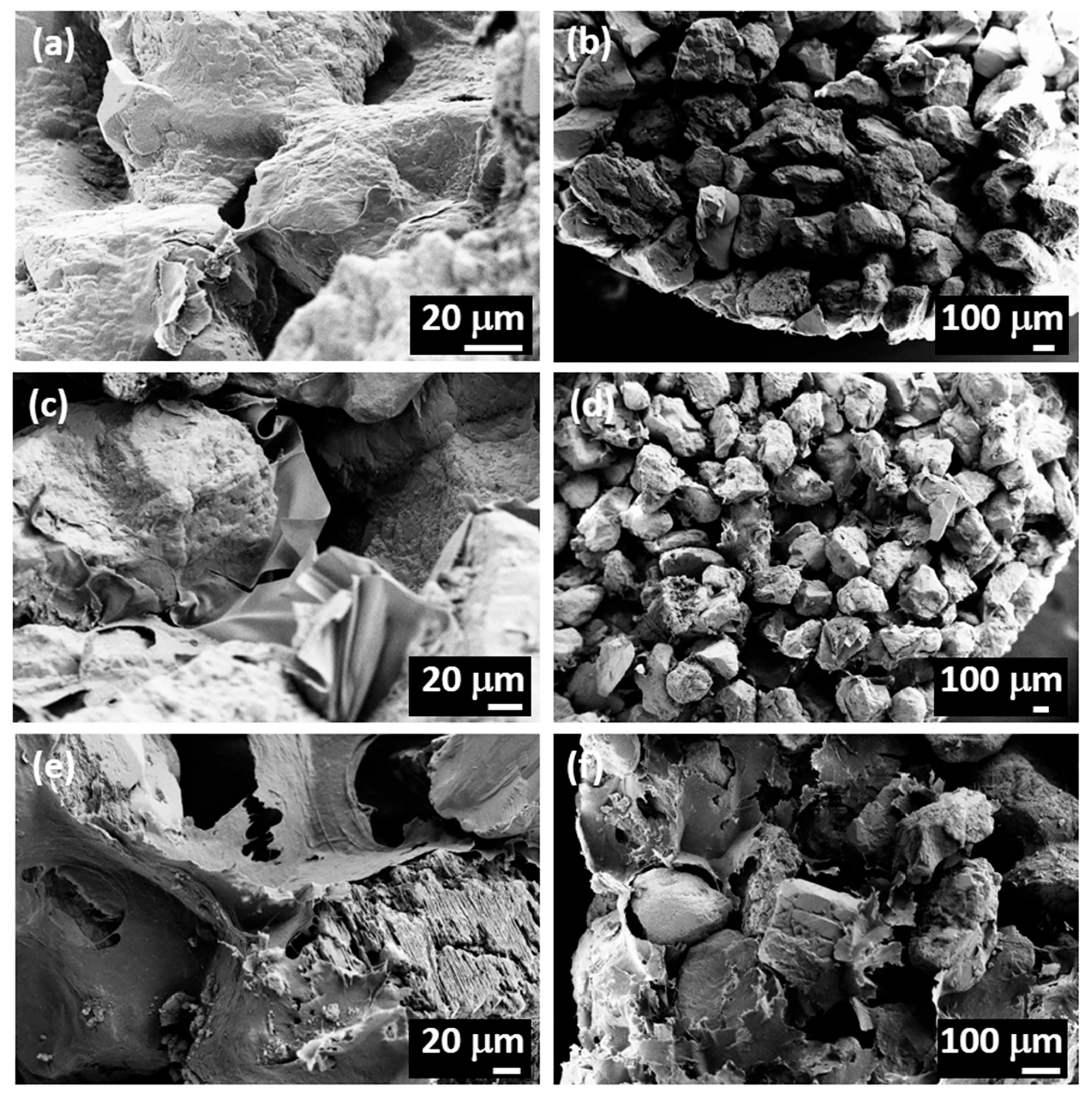
Scanning electron microscopy images of the pellets formed with (a, b) CMC_0.09_, (c, d) CMC_0.7_, and (e, f) CNF (2X).

KP and BKP presented continuous plastic deformation (Figure 2a as well as Figure S3a, KP 6X) while HPC, methylcellulose, and CNF showed semiplastic deformation followed by large cluster formation upon fracture (Figure 2b,c as well as Figure S3b, CNF 2X and HPC, respectively). The remaining composites fractured into large (CMC_0.7_) or small clusters (CMC_0.09_, pectin) above a critical load (Figure 2d,e as well as Figure S3c). When considering the ultimate compression strength (UCS) that was sustained by the pellets, fracture type ii showed the largest values, typically at ca. 10% strain. For fractures of type i, no catastrophic failure was observed. This difference in behavior can be attributed to (a) the differences in the conformation of the network of carbohydrate polymers formed between grains, larger clusters representing the formation of larger cohesive networks; (b) the cohesive interactions within the amendment’s network, i.e., polymer–polymer interactions; and (c) the adhesive interactions of the polymer at the sand’s interface. Typically, plastic deformation corresponds to substantial slippage at the grain–polymer/fiber interface,^30^ while cluster size depends on how homogeneous the polymer–sand interaction within the composite is, which is a result of capillary gelation within the grains’ network during consolidation, i.e., during drying. As depicted in Figures 3 and S4, when the polymer solution gels at a low concentration, it stretched within the confined space. In contrast, polymers that gel at high concentrations migrated and created capillaries between sand grains before consolidating. For some polymers such as chitosan, consolidation and adhesion occured simultaneously on the surface of the grains and at the contact point of the grains. These observations were also previously observed when drying polysaccharide solutions under planar confinements, i.e., between plates.^34^

### Morphological Analysis

Based on the fracture behaviors, three samples with distinct properties were more closely assessed to associate internal structure with the mechanical responses observed. These samples, CNF (2X), CMC_0.09_, and CMC_0.7_, were observed under scanning electron microscopy. As expected, because the concentration at which gelation occurs differs between the different organic amendments, the internal structures formed were substantially different (Figure 3). Previous observations suggest that late gelation would favor a higher concentration of the polymer within small capillaries formed at the contact points of the sand granule. In contrast, an early gelation of the polymer solution would result in a homogeneous contraction of the gelled phase across the consolidating pellets, and therefore the polymer would be homogeneously present within pores between granules.^34,35^ Lastly, in the case of fibers where gelation at low concentration is typically observed (ca. 1.5 wt %), the impact of shear thinning is expected to alter the network orientation within the pores.^34^ Indeed, in the case of CNFs, a honeycomb-like network of fibers was observed wrapped around the sand granules. This is similar to previous reports when compositing CNFs with grains considerably larger than the fibrils.^30^ The open faces of the honeycomb structure suggest that the concentration was not sufficient to lead to an early gelation of the dispersion, that the fibers consolidated at a later stage, or that the high shear in the faces led to the concentration of CNFs within the plateau border. In the case of CMC_0.09_, the polymer phase was not clearly differentiated from the sand grains, only in some areas as highlighted in Figure 3a. In some areas, small polymeric bridges that may contain high polymer concentration were observed near the contact points. Possibly, the consolidating polymer gelled at rather high concentrations and migrated toward capillary bridges between granules prior to consolidation. This contrasts with CMC_0.7_, which was infiltrated into the sand as a thick gel. Thereafter, during consolidation, the gel consolidated into thin films observable between the grains. These sheets were visible across the full cross section shown in Figure 3d. Some bridges could also be observed forming between the contact points of the granules (Figure 3).

### Dynamic Vapor Sorption Analysis

**Figure 4:**
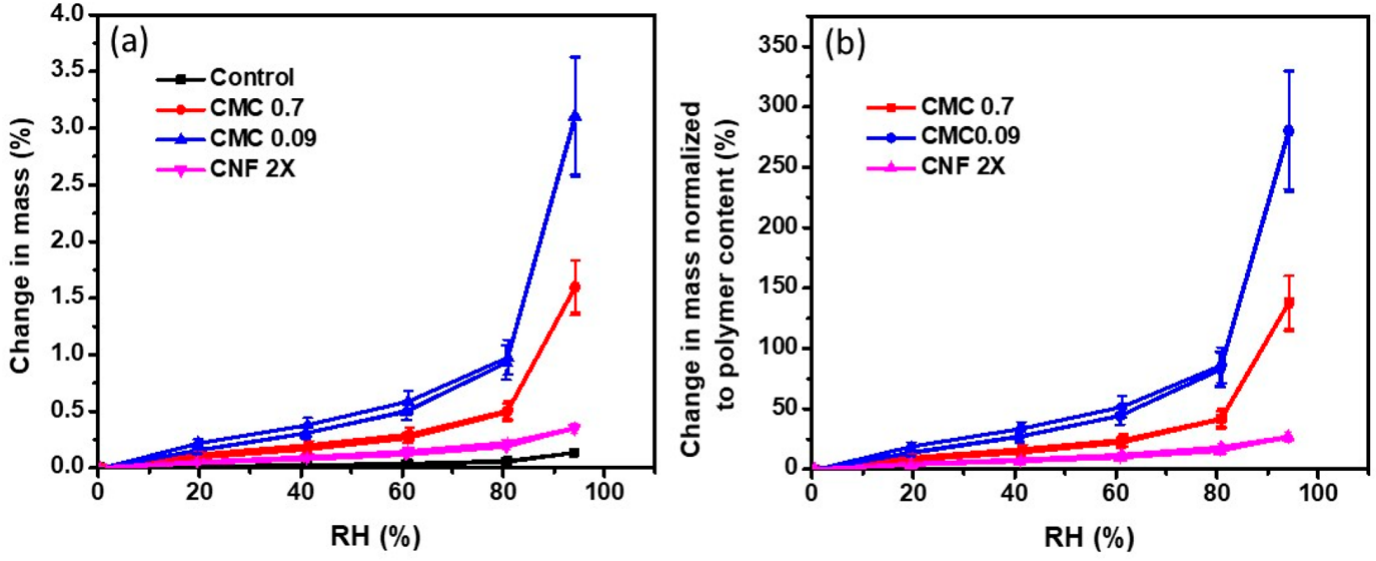
(a) Changes in mass for the composite pellets as a function of relative humidity. (b) Mass change normalized to the polymer content after subtraction of the corresponding change in mass for the control.

Dynamic vapor sorption was performed on the samples described in Figure 4. Considering that the added fraction of biomacromolecules in the composites was small (1.06 wt % (CMCs) or 2.12 wt % (CNF)), the difference was remarkable when compared with the packed sand granules alone (Figure 4a, control), which emphasizes that the macromolecules’ specific interactions with water were responsible for the hygroscopic response. This is more clearly highlighted when the water adsorption is normalized to the polymer content (Figure 4b). CMC_0.09_, CMC_0.7_, and CNF 2X adsorbed nearly 300%, 150%, and 25% of their weight at 95% RH. This is surprising and may suggest that CMC may contribute to an increased capillary condensation, potentially associated with its localization toward contact points where capillary condensation occurs first. A slight increase in mass (<0.1%) was observed for the granules without bio-based amendments above 80% RH as associated with capillary condensation. A continuous increase in mass was observed until 80% RH, after which a large increase took place until 95% RH.

**Figure 5:**
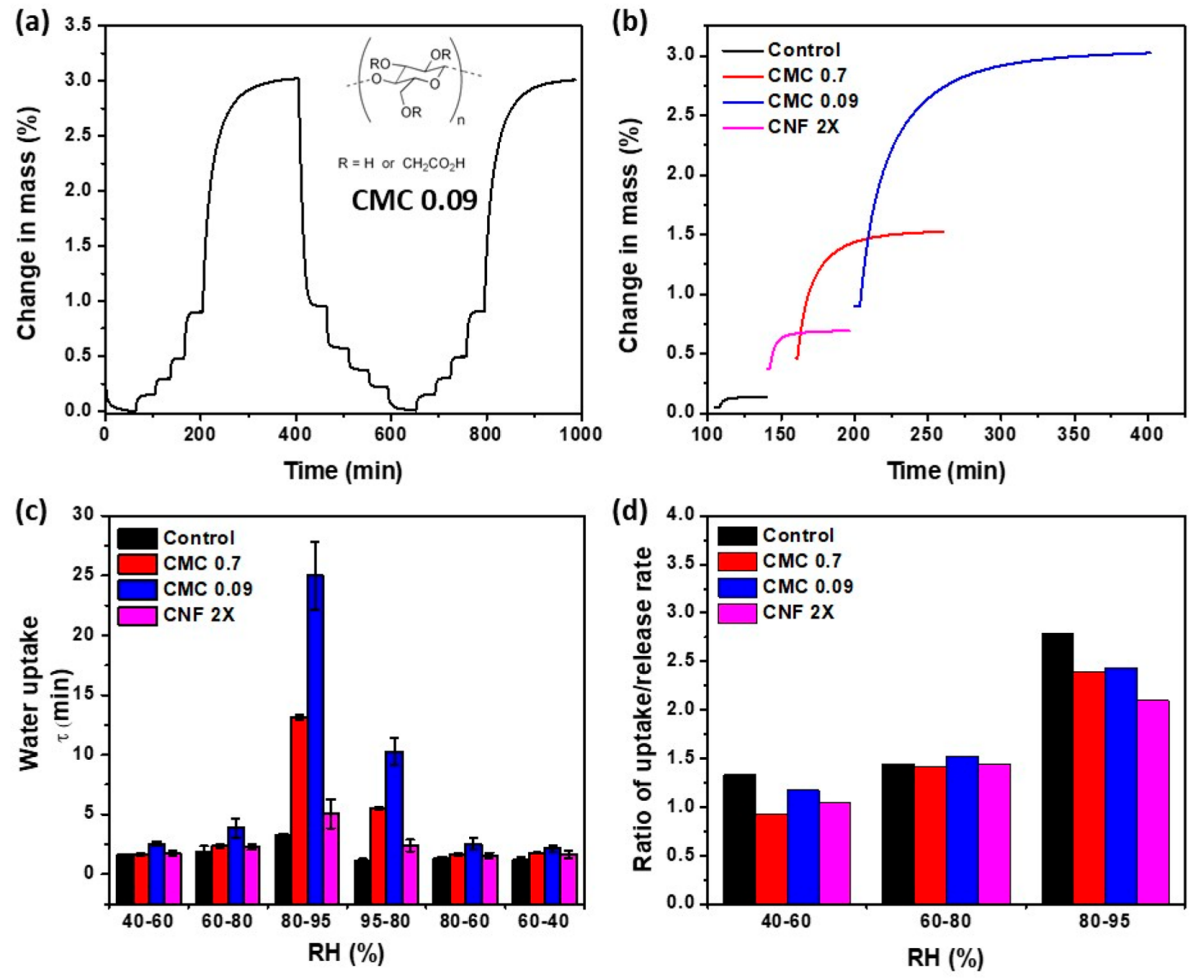
(a) Representative water adsorption isotherm from pellets consolidated with CMC_0.09_. (b) Representative water adsorption isotherms for the 80–95% RH transition in pellets containing CMC_0.7_, CMC_0.09_, CNF 2X, or no amendments (control sand). (c) Water uptake constant or step response constant corresponding to the time required to reach 63.2% ((1 – 1/*e*) × 100%) of the plateaued isotherm. (d) Corresponding ratio of uptake to release rates (higher ratio indicating slower uptake).

The dynamics of water interactions were evaluated for CMC_0.09_, CMC_0.7_, and CNF 2X. An example of dynamic water vapor sorption isotherms is shown in Figure 5a for CMC_0.09_. As can be seen, the dynamics were not proportional between each humidity transition as well as between uptake and release. Release was typically faster than uptake, and higher humidity resulted in much slower uptake isotherms, as also associated with higher uptake quantities. Representative differences for the transition from 80% to 95% RH are shown in Figure 5b for the three cellulosic fibers and the control in the absence of polymer. As can be observed, the increase in total quantity adsorbed resulted in significant shifts in the characteristic adsorption time constant. The water uptake/release constant was then extracted by obtaining the time required to reach 63.2% of the plateau value for each transition (Figure 5c). For all samples, the water release was considerably faster compared to uptake in the 80–95% transition with the most pronounced differences being in the order of CMC_0.09_ > CMC_0.7_ > control > CNF 2X. The water release was also faster than uptake for the 60–80% and 40–60% transitions except for the 40–60% transition of CMC_0.7_. Overall, the release rate was substantially higher than the uptake rate of water, and the trend was more pronounced for larger RH transitions (Figure 5d).

### Quantitative Analysis of Mechanical Responses

**Figure 6:**
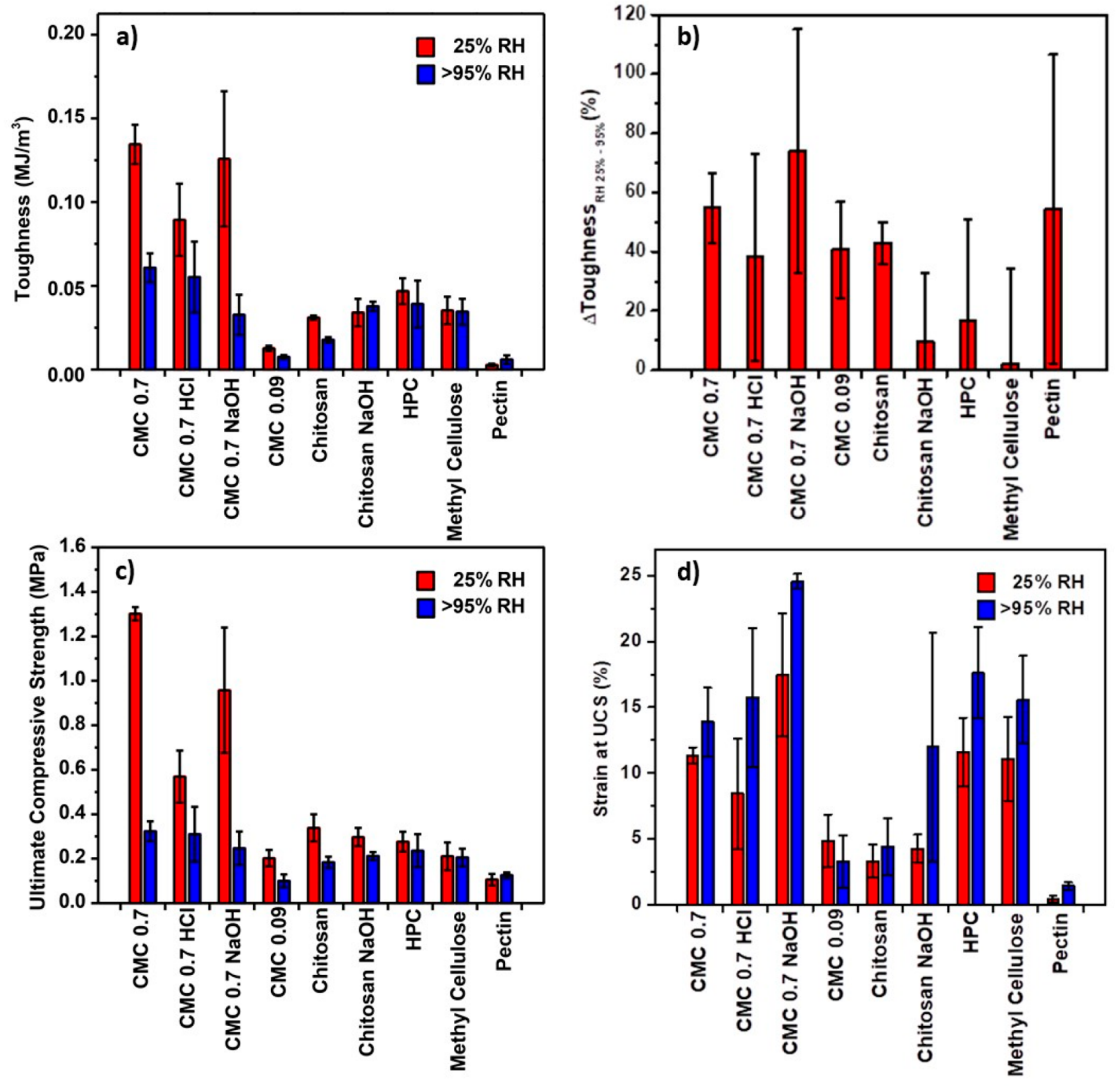
(a) Toughness (in MJ m^–3^) for the pellets as a function of carbohydrate polymers added after a 25% strain. (b) Relative change in average toughness resulting for samples exposed to 25% RH or >95% RH prior to compression. (c) Corresponding ultimate compressive strength (UCS). (d) Strain at UCS.

The compressive response of the pellets was quantified for overall toughness, ultimate compressive strength (UCS), and strain at failure (Figure 6). Of note, toughness may be considered more indicative of the consolidating potential of the polymers as it is less affected by early failure due to defects, which can typically occur in brittle materials.

When considering the full data set as qualitatively described in Figure 2, including preliminary tests on fibers, the overall toughness at 25% humidity was the best for CMC_0.7_ (0.134 MJ m^–3^) followed by CNF6P 2X (0.097 MJ m^–3^) and by a set of natural polymers with similar performance formed by chitosan (0.031 MJ m^–3^), HPC (0.047 MJ m^–3^), BKP 2X (0.038 MJ m^–3^), and methylcellulose (0.035 MJ m^–3^). CMC_0.09_ (0.013 MJ m^–3^), KP 6X (0.004 MJ m^–3^), and pectin (0.003 MJ m^–3^) presented the worst performance. Interestingly, BKP showed a substantially larger increase in toughness than KP, which could be associated with the fact that BKP was never dried and KP was once-dried (in contrast to “never-dried” fibers), leading to the presence of smaller flocs during assembly. Given that KP and BKP required an excess to provide sufficient cohesion, the samples were not looked into further. Instead, polymeric and nanofibrous systems were explored more systematically.

A similar trend was observed for the UCS as reported for toughness, implying that toughness was predominantly influenced by the initial response to compression up to the maximum stress, with postfracture contributions likely being less critical to the overall toughness. When examining polymeric systems exclusively, it is evident that the carboxymethyl functional group significantly enhanced performance, as depicted in Figure 6. Larger molecular weights also resulted in better performance, potentially due to their larger network when compared to lower molecular weight polymers. The fact that CMC performed better than chitosan may be ascribed to the early coating of chitosan onto the granules rather than between granules at the consolidation points (the three different types of interactions are showcased in Figure S4). When compared with CMC_0.09_, CMC_0.7_ had an over 10-fold improvement in toughness at 25% RH. This implies that migration of the polymer, as associated with late gelation as is the case of CMC_0.09_, significantly reduced the overall cohesion of the pellet.

The UCS and strain at UCS of different pellets are shown in Figures 6c and 6d, respectively. The pellets with minimal strain at failure, such as CMC_0.7_, showed high strength, showing their proportional brittleness as expected. This phenomenon can be explained by a strong interaction between the polymer and the grains combined with a brittle response of the CMC as a binder, which results in little plastic deformation prior to failure.

The polymers were also evaluated at different pHs during assembly to further assess the impact of charges on functional groups on the properties of the composites obtained. In the case of chitosan, they were assembled at pH 5 or subsequently exposed to pH 13 (chitosan NaOH), where chitosan does not have any cationic charge and has poorer water interactions. CMC was assessed whether it was assembled in distilled water (carboxylate to carboxylic acids ratio ∼50%) or subsequently exposed to pH 1 (∼100% carboxylic acids, CMC_0.7_ HCl) or pH 13 (∼100% carboxylates, CMC_0.7_ NaOH). At an RH of 25%, when CMC is predominantly in either its carboxylate salt or carboxylic acid form, there was a small alteration in cohesion. Specifically, there was approximately a 10% decrease in toughness when CMC was in its carboxylate salt form and about a 33% decrease in toughness when it was in its carboxylic acid form, as compared to an intermediate state of charge. This unexpected change highlights the significance of the interfacial chemistry of the sands, which could also be influenced by the presence of NaOH or HCl. No significant change was observed (∼9%) in toughness for chitosan in the presence of NaOH when compared with fully charged chitosan, further confirming that a secondary phenomenon that depends on the surface chemistry of the sand may happen in parallel. The overall relatively small differences in mechanics caused by the changes in the charged state of these polymers could suggest that the original conformation of assembly is more critical than subsequent ion exchanges. When further considering the case of CMC_0.7_, at 25% RH, the UCS was considerably higher in the absence of pH changes compared to the uncharged CMC obtained in acidic conditions. This suggests that the charges on CMCs do lead to either a more cohesive CMC–CMC network or higher CMC–sand interactions.

An increase in humidity, from 25% to over 95%, significantly reduced the overall toughness of all charged and highly polar polymeric systems, while most uncharged systems (including chitosan NaOH and CMC HCl) exhibited considerably less pronounced changes. Additionally, when increasing the humidity, a substantial but relatively diminished effect was observed on both the UCS and the strain at which UCS occurred for all of the polymers. At high humidity, both chitosan and CMC_0.09_ composites exhibited similar compression responses, whereas CMC_0.7_ composites displayed notably greater plasticity under compression, suggesting increased sand–polymer interface slippage for the latter. This is likely to be associated with the increased competitive interactions at the sand–polymer interface and between polymers and water molecules. A decrease in toughness of ca. 40% for the case of CMC_0.09_ and chitosan was observed while a decrease above 50% was observed for CMC_0.7_. For uncharged polymers such as methyl cellulose and HPC, a small reduction (<20%) in toughness was observed at higher humidity. Figure 6b highlights the relative change in average toughness due to changes in humidity and emphasizes that CMC_0.7_ had the most significant decrease in toughness upon increasing the humidity. The same trend can be seen for the differences in UCS as a function of the humidity (Figure 6c). Surprisingly, all polymers, with the exception of CMC_0.09_, had an increased strain at UCS at higher humidity (Figure 6d). While the overall strength and toughness were reduced more for charged polymers, the strain at a maximum load was higher for all systems. This suggests that the hygroscopic nature of these polysaccharides had an impact on the plasticity of all systems. Overall, it appears that a balance between the mechanical properties of the networks formed by the polysaccharides in soils and water dynamics should be considered.

**Figure 7:**
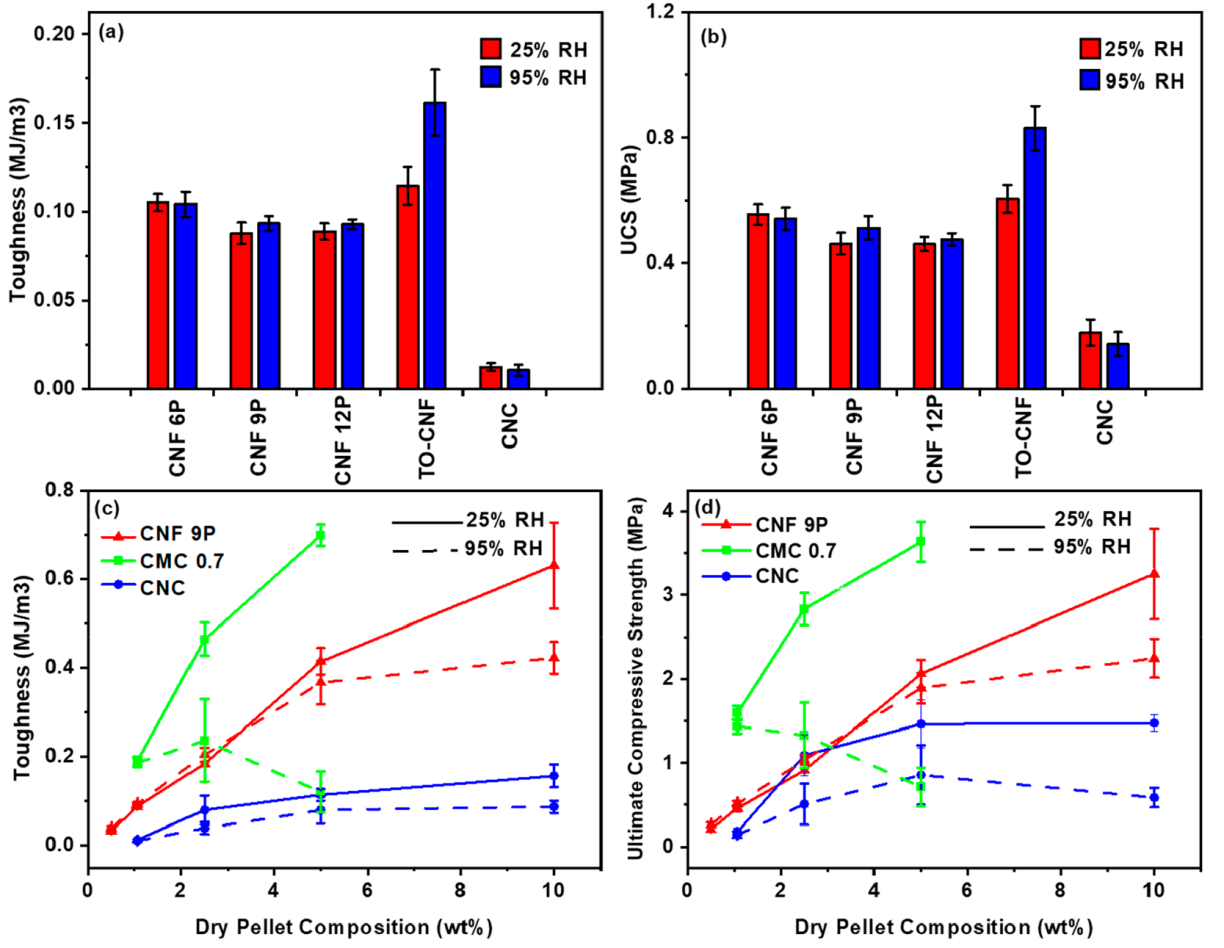
(a) Toughness in MJ m^–3^ for 1.06% dry-weight nanocellulose in the pellets. (b) Ultimate compressive strength (UCS) for the pellets as a function of added nanocellulose after a 25% strain and 1.06% dry weight nanocellulose in the pellets. (c) Toughness in MJ m^–3^ as a function of polymer composition in the pellets. (d) Ultimate compressive strength (UCS) for the pellets as a function of dry weight percentage of nanocellulose after a 25% strain.

Nanofibers, including various types of cellulosic fibers and nanocrystals with or without surface modifications and different levels of fibrillation severity (from 6 to 12 fibrillation passes), were assessed as binders in sand composite pellets at various concentrations, as illustrated in Figure 7. In general, the fibers presented a lower toughness than the dissolved macromolecules; however, the strain at the UCS was typically comparable with the better polymeric performers such as CMC_0.7_, HPC, or methylcellulose (Figures 6d and S5). This highlights the difference between slippage imparted by fibers compared to the proportionally more brittle fractures observed by most macromolecules. For finer fibrils (TO-CNFs), finer fibrils (TO-CNFs),^29^ enhanced toughness and strength of composites were observed compared to mechanically fibrillated systems, indicating that the oxidation process, and subsequent fibrillation to elementary fibrils sizes improved the mechanical properties of the composited sand pellets (Figure 7a,b). Interestingly, for mechanically fibrillated CNFs, the degree of fibrillation had little influence on the mechanical properties beyond 6 fibrillation passes. This corroborates with previous observations on a size-dependent threshold existing for particle–fiber interactions, where no benefits are gained from smaller fibers, depending on particle sizes.^30^ In comparison to the other sand pellets, CNCs had the lowest mechanical properties, which is possibly attributed to their smaller aspect ratio as well as the brittle nature of their assemblies. Adding more CNF or CNC to the sand mixture increased both toughness and strength, which was likely due to improved interactions between the nanocellulose and the grains in the pellets and to an improved nanocellulosic network across the pores. The trend followed a sigmoidal curve, tending to an upper threshold. Above 2.5%, the benefits of increased content of CNCs were rather small. The upper threshold was not as markedly reached for CNF9P at 10%. For CNFs and CNCs, the humidity increase to >95% RH from 25% RH had little effect on the mechanics except at a content of 10% of fibers/crystals, where the difference was more marked. CMC_0.7_ was also evaluated as a function of concentration, serving as a reference point between polymeric and nanocellulosic systems. A similar trend was observed for CMC at higher upper values for toughness and UCS at 25% RH, though the upper threshold was not reached at 5% of CMC_0.7_. Higher contents could not be used for CMC_0.7_ as a very thick, rubber-like gel was obtained at 5%. CMC_0.7_ demonstrated a sharp, concentration-dependent decrease in toughness and compressive strength at a humidity >95% beyond a content of 2.5 wt %. This is potentially a result of significant water adsorption, which led to the formation of a hydrogel-like material with poor mechanics. Therefore, the addition of nanocellulose offers the potential for improving the mechanical properties of sand pellets without compromising the strength at high humidity when compared with CMCs. Overall, optimizing the performance of these cellulosic biomaterials toward optimized formulations will require careful evaluation of the surface functional groups, solid fraction, molecular weights, and morphology. Possibly, formulations considering nanocelluloses and polymers mixtures may yield improved overall results.

This study explored diverse functional interactions between various biopolymers and quartz particles to improve the mechanical properties and water interactions of sandy soils. Some of the results from the existing literature on cellulose–mineral interactions may be extrapolated to design future experiments. For example, a large body of literature is dedicated to exploiting the intimate interactions between clays or mineral particles with nanocellulose, e.g., in forming strong and tough nanocomposites.^36−38^ Typical interactions are driven by secondary interactions, though these may be rather complex in the case of a multicrystalline material such as quartz.

As mentioned above, the utilization of polysaccharides for sand amendment is a promising avenue for enhancing the cohesion and water interactions in sandy soils. Financial viability and off-course environmental costs of processing and amending soils will be another key factor behind their implementations, for example, in arid areas. CMC is typically obtained from dissolving grade pulp and presents a relatively high cost. Although CNFs should have lower costs when benefiting from the economy of scale, a compromise may be made, and mixed compositions may be more favorable for implementation. As commonly observed with nanocellulose, the development of efficient processing routes will be pivotal in optimizing the costs. Our focus extends to cellulose derivatives and other polysaccharides, aligning with the need for sustainable alternatives to address key issues in conventional sand. Moreover, the inclusion of HPC, chitosan, pectin, and sodium alginate adds versatility to the approach, catering to specific soil conditions. Emphasizing sustainability, we stress the importance of locally sourced materials to reduce transportation impact and advocate for minimally exhaustive processes to mitigate environmental hazards. These considerations are paramount in optimizing the overall sustainability and long-term effectiveness of the proposed strategy, aligning with the imperative to improve desertic soil cohesion for enhanced plant growth and cropping potential over extended periods. Thereafter, biomass collection and management through local, national, and global governance may prove to be key to the success of the endeavors associated with the results put forward herein.

## Conclusion

In this study, a wide range of polysaccharide-based amendments, in various physicochemical forms, including dissolved polymers, nanofibrils, and pulp fibers, were evaluated for their potential as amendments for desertic soil. Insights into their impact as sand amendments are provided:

1. Cohesive force, resistance to erosion, and internal architecture: dissolved polymers exhibited the highest cohesive forces within the composites, followed by nanofibrils and pulp fibers. The network of fibers, typically formed by larger polymers and nanofibrils, enabled the creation of sheets within the porous network and larger clusters, contributing to better long-term erosion resistance and enhanced compression resistance, especially when compared to smaller polymers. Among fibers, TO-CNFs notably enhanced both strength and toughness due to their rheological properties, allowing for the formation of robust fluid slurries even at higher concentrations. While the mechanics of composites containing dissolved polymers were negatively affected by humidity, those containing nanofibrils were mostly unaffected.
2. Ability of the system to uptake and release water: lower molecular mass polymers showed faster and larger hygroscopic response than other systems, with variations in uptake and release rates among the samples, particularly in higher relative humidity transitions, suggesting potential contributions to capillary condensation.
3. Applicability of the methodology put forward herein: the proposed framework is straightforward and easily reproduced toward rapid benchmarking of carbohydrate polymers as amendments to desertic soils.

Overall, considering the fundamental aspects of this study, optimizing the performance of carbohydrate polymers toward optimized formulations will require careful evaluation of the surface functional groups, solid fraction, molecular weights, and morphology for their ability to imbue cohesion to networks of large grains. Potentially, a polymer/fiber-grain cluster size could be attributed to the periodic failure observed during compression (e.g., as seen in Figure 2d). Several studies have attempted to correlate the compression response of such packed grains with packing, interfacial interactions, and stress distribution.^30,39^ However, more practical data generation and benchmarking may enable a faster approach to a full understanding of the system.

With regard to the long-term goals associated with this study, i.e., addressing sand mobility and associated desertification, we expect that further property mapping between sand dimensions as well as surface chemistry combined with the possible polymers and fibers obtained from food waste will become available from the proposed platform. More advanced models will consider packing of the sand grains, sustainability of the obtained biopolymers (in terms of processing costs and considering other valorization streams competing with amendments), and long-term resistance to erosion due to polymer degradation or migration. A cross-correlation between regionally available biomass, processing costs, and performance can enable decision-making toward consolidation and regeneration of desertic soils, thus enhancing the chances of a functioning bioeconomy in arid areas.^13^

## Supporting Information Available

Plots of toughness and UCS for CMC_0.7_ with varying drying conditions (Figure S1), image of failed pellets (disks) obtained using alginate as an amendment (Figure S2), representative schematics for distinct fracture behaviors of pellets (Figure S3), schematics highlighting the effect of gelation point on the formation of distinct microstructures between the grains during drying (Figure S4), plots of strain at UCS for the pellets as a function of added nanocellulose after a 25% strain and 1.06% dry weight nanocellulose in the pellets (Figure S5) (PDF)
