# Supplementary material for: Benchmarking the Humidity-Dependent Mechanical Response of (Nano)fibrillated Cellulose and Dissolved Polysaccharides as Sustainable Sand Amendments
Source: Biomacromolecules. 2024 Mar 8;25(4):2367–77. doi: 10.1021/acs.biomac.3c01294 (PMC11005006; doi:10.1021/acs.biomac.3c01294)
Supplement: Supplementary file 1 — bm3c01294_si_001.pdf [file bm3c01294_si_001.pdf]

SUPPLEMENTARY INFORMATION

Benchmarking the humidity-dependent mechanical response of (nano)fibrillated cellulose and dissolved polysaccharides as sustainable sand amendments

*M-Haidar A. Dali[a,b,±], Roozbeh Abidnejad[c,±], Mohamed Hamid Salim[a,b,d,±], Mamata Bhattarai[c], Monireh Imani[c], Orlando J. Rojas[c,f], Luiz G. Greca[g], Blaise L. Tardy[a,b,d]**

a. *Khalifa University, Department of Chemical Engineering, Abu Dhabi, United Arab Emirates*
b. *Research and Innovation Center on CO2 and Hydrogen, Khalifa University, Abu Dhabi, United Arab Emirates*
c. *Department of Bioproducts and Biosystems, School of Chemical Engineering, Aalto University, P. O. Box 16300, FI-00076 AALTO, Finland*
d. *Center for Membrane and Advanced Water Technology, Khalifa University, Abu Dhabi, United Arab Emirates*
e. *Bioproducts Institute, Department of Chemical and Biological Engineering, Department of Chemistry and Department of Wood Science, University of British Columbia, 2360 East Mall, Vancouver, BC V6T 1Z4, Canada.*
f. *Laboratory for Cellulose & Wood Materials, Empa—Swiss Federal Laboratories for Materials Science and Technology, Überlandstrasse 129, 8600 Dübendorf, Switzerland*

± These authors contributed equally

* To whom correspondence should be addressed

E-mail: blaise.tardy@ku.ac.ae

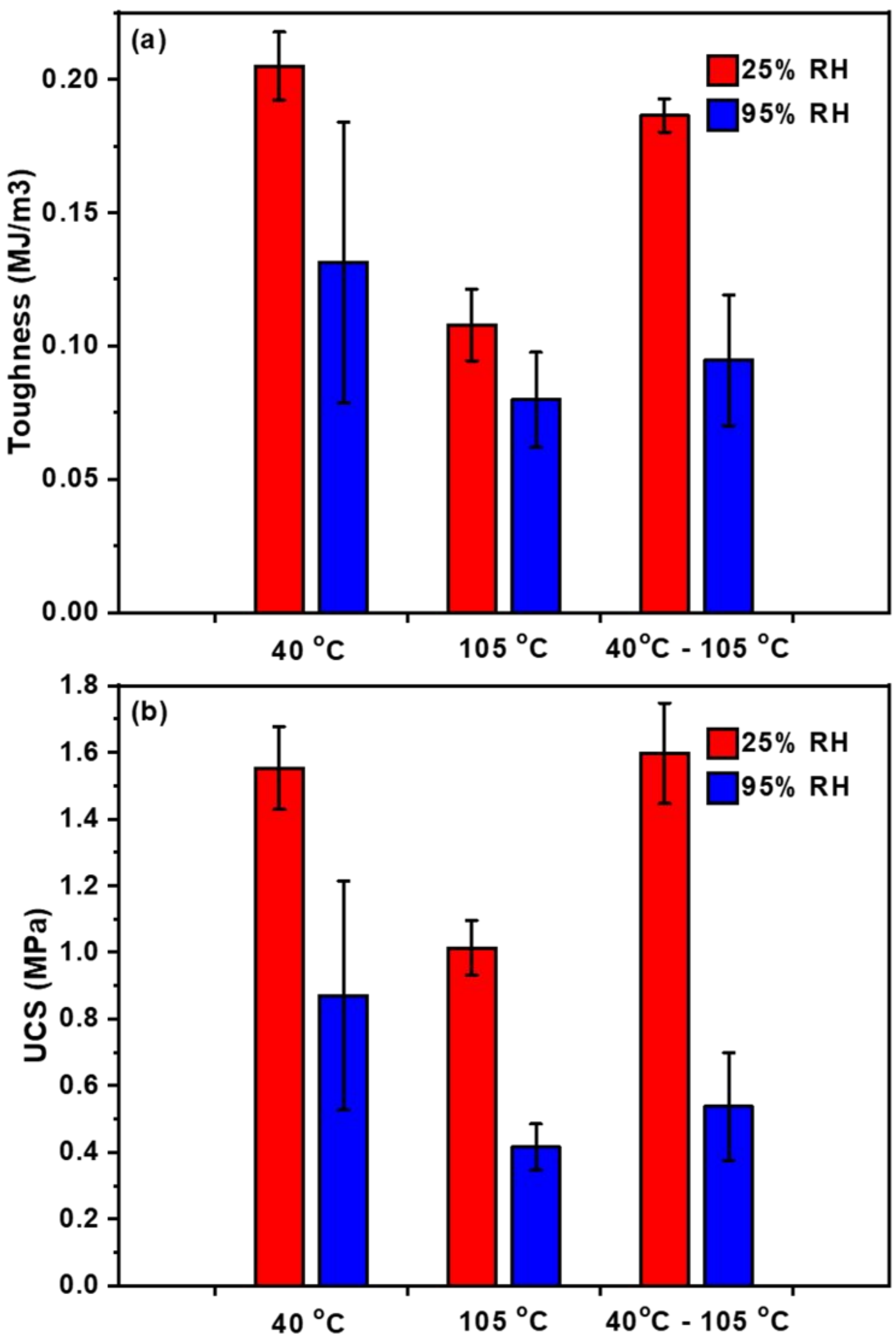

Figure S1 Top- Toughness of the pellets as a function of RH and drying conditions at varied drying temperatures for $CMC_{0.7}$ and, Bottom – corresponding UCS.

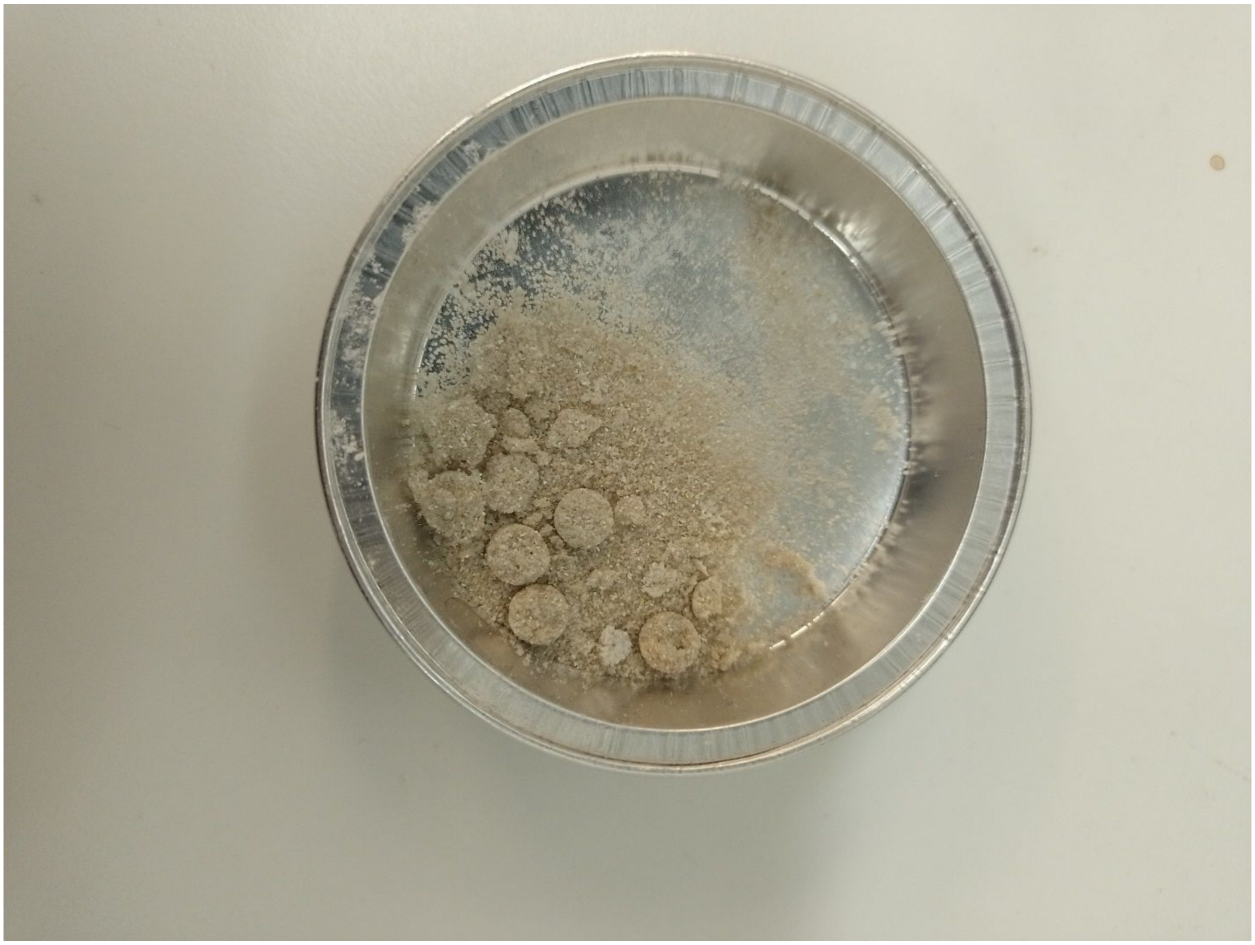

Figure S2 Representative crumbled sand-alginate cylinders (herein disks) obtained as a result of alginate migration to the top, open side of the mold during consolidation.

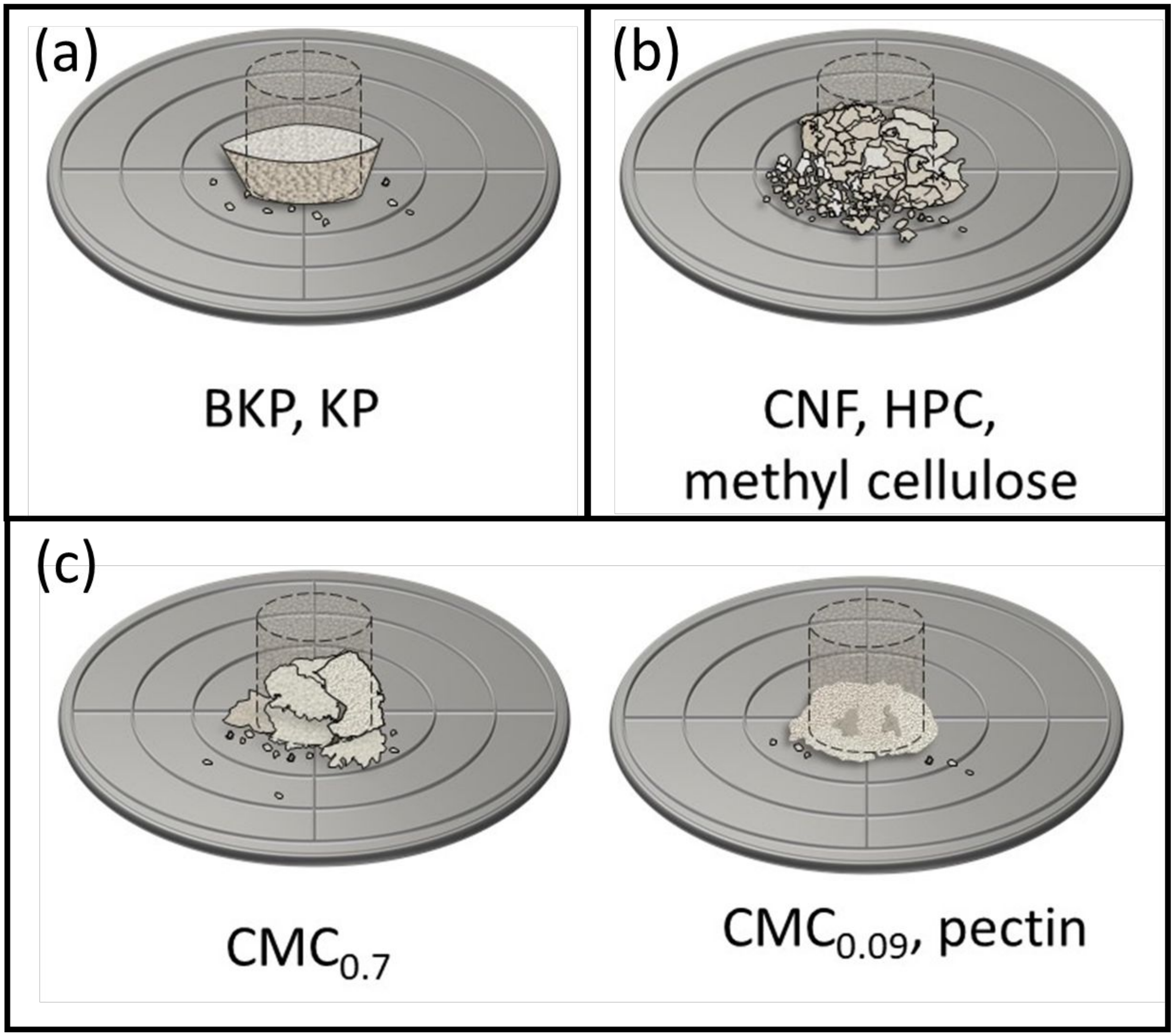

Figure S3 Representative response to compression (fracture behavior) at 25% RH.

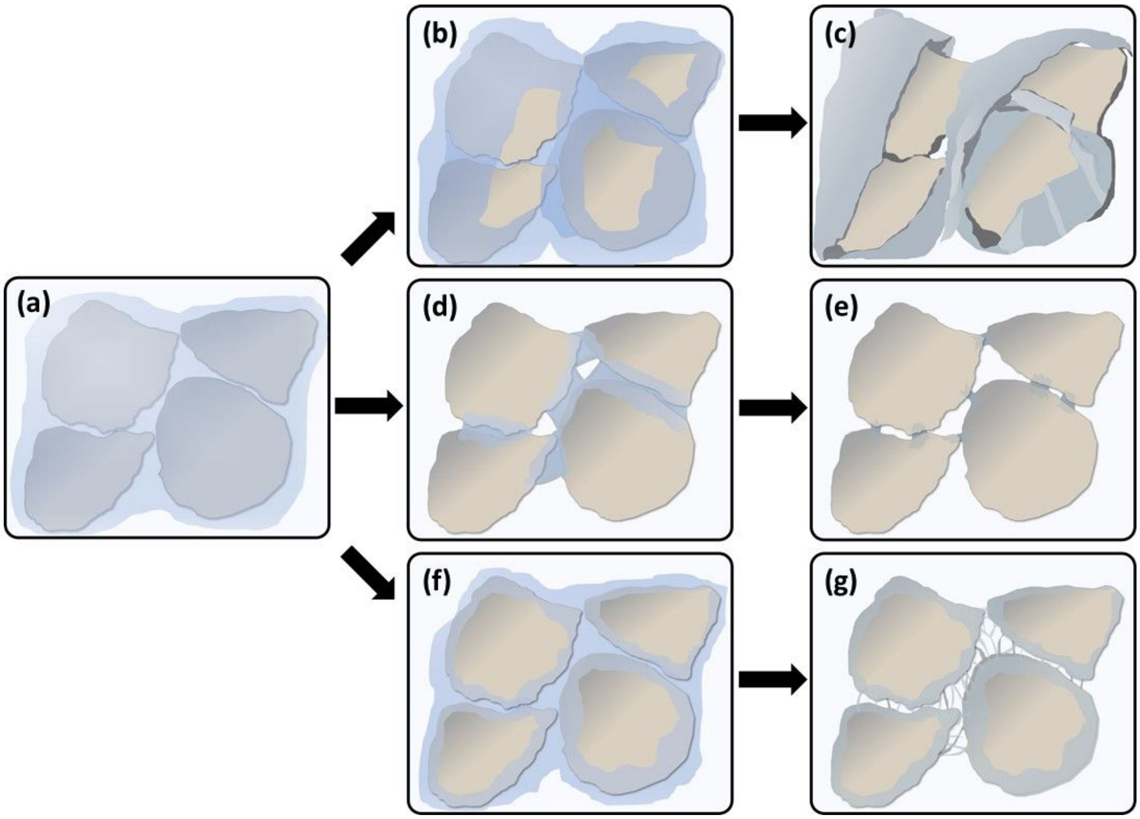

Figure S4 the interactions between sand granules and polymer phases during the drying process: (a) Initial mixture state. (b and c) Drying with a polymer gelling at a low concentration, and after consolidation primarily through the formation of intergranular polymer sheets, respectively. (d and e) Drying with a polymer gelling at a high concentration, and after consolidation mainly through small capillaries following migration to capillaries between grain contact points, respectively. (f and g) Drying with chitosan, and after consolidation mainly through very thin and numerous capillaries following migration to capillaries between grain contact points, respectively.

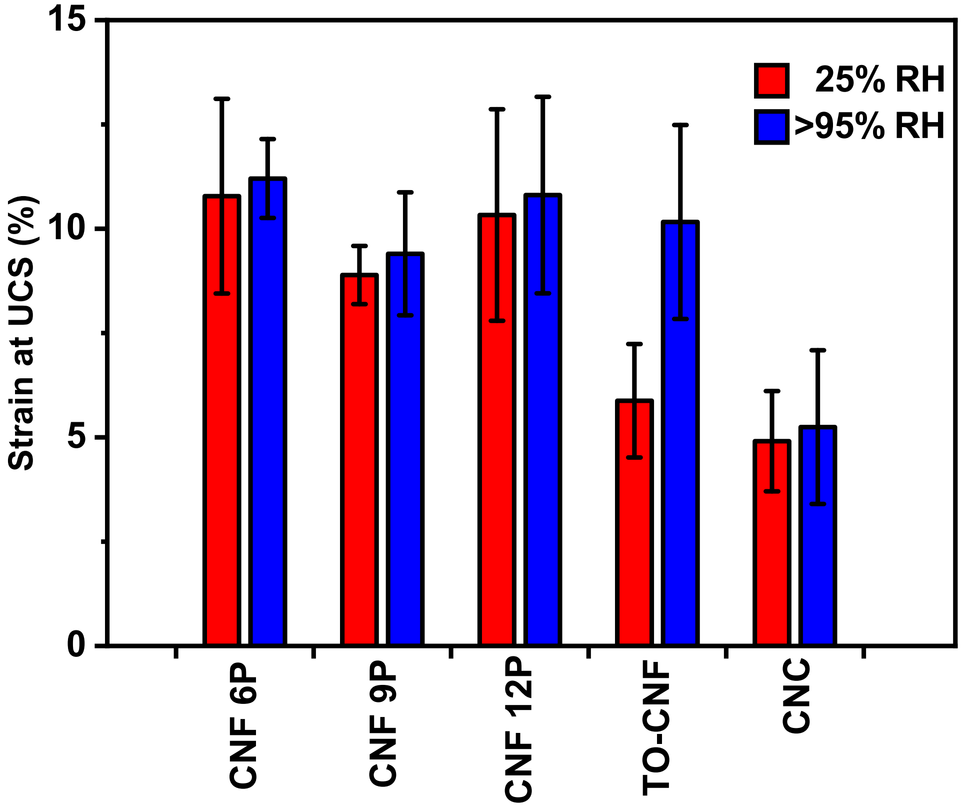

Figure S5 Strain at ultimate compressive strength (UCS) for the pellets as a function of added nanocellulose after a 25% strain and 1.06% dry weight nanocellulose in the pellets
